# Kilogram-scale production of strong and smart cellulosic fibers featuring unidirectional fibril alignment

**DOI:** 10.1093/nsr/nwae270

**Published:** 2024-08-05

**Authors:** Jianguo Li, Chaoji Chen, Qiongyu Chen, Zhihan Li, Shaoliang Xiao, Jinlong Gao, Shuaiming He, Zhiwei Lin, Hu Tang, Teng Li, Liangbing Hu

**Affiliations:** Department of Materials Science and Engineering, University of Maryland, College Park, MD 20742, USA; Department of Materials Science and Engineering, University of Maryland, College Park, MD 20742, USA; Department of Mechanical Engineering, University of Maryland, College Park, MD 20742, USA; Department of Materials Science and Engineering, University of Maryland, College Park, MD 20742, USA; Department of Materials Science and Engineering, University of Maryland, College Park, MD 20742, USA; Department of Materials Science and Engineering, University of Maryland, College Park, MD 20742, USA; Department of Materials Science and Engineering, University of Maryland, College Park, MD 20742, USA; Department of Materials Science and Engineering, University of Maryland, College Park, MD 20742, USA; Department of Materials Science and Engineering, University of Maryland, College Park, MD 20742, USA; Department of Mechanical Engineering, University of Maryland, College Park, MD 20742, USA; Department of Materials Science and Engineering, University of Maryland, College Park, MD 20742, USA

**Keywords:** cellulosic fiber, uniaxial fibril, biomass, actuator, smart fiber

## Abstract

Multifunctional fibers with high mechanical strength enable advanced applications of smart textiles, robotics, and biomedicine. Herein, we reported a one-step degumming method to fabricate strong, stiff, and humidity-responsive smart cellulosic fibers from abundant natural grass. The facile process involves partially removing lignin and hemicellulose functioning as glue in grass, which leads to the separation of vessels, parenchymal cells, and cellulosic fibers, where cellulosic fibers are manufactured at kilogram scale. The resulting fibers show dense and unidirectional fibril structure at both micro- and nano-scales, which demonstrate high tensile strength of ∼0.9 GPa and Young's modulus of 72 GPa, being 13- and 14-times higher than original grass. Inspired by stretchable plant tendrils, we developed a humidity-responsive actuator by engineering cellulosic fibers into the spring-like structures, presenting superior response rate and lifting capability. These strong and smart cellulosic fibers can be manufactured at large scale with low cost, representing promising a fiber material derived from renewable and sustainable biomass.

## Teaser

A strong and smart cellulosic fiber is fabricated from fast-growing grass material at kilogram scale, which features an interesting humidity-responsive functionality with superior response rate and lifting capability, representing a promising candidate for next-generation sustainable functional fibers.

## INTRODUCTION

Functional fibers are ubiquitous in our society, used in a range of applications, such as flexible fibers for textiles [1–3], stiff and strong fibers for construction [4,5], hydrophobic/hydrophilic fibers for solution purification [6–8], and conductive fibers for energy storage and conversion [9–12]. Various raw materials have been used for such applications, including fibers made of carbon [13–16], synthetic polymers [17–20], metals and alloys [21,22], and ceramics [23,24]. For example, carbon-based fibers generally exhibit high mechanical strength (1–4 GPa in tensile strength), high thermal (500–3000 W m^−1^ K^−1^) and electronic conductivities (1–10 × 10^5^ S m^−1^), making them attractive for applications relating to lightweight vehicles, intelligent actuators, additive manufacturing, and electronic products. However, the manufacturing process of carbon-based fibers generally involves high-temperature (oxidation or pyrolysis) and complex treatments, resulting in high production costs as well as potential environmental issues. Similarly, synthetic polymer fibers demonstrate some advantageous features, such as high mechanical strength and ease of manufacturing, but they are commonly made of nonrenewable petroleum-based resources and can be difficult to degrade at the end of their product lifetime, which can lead to severe plastic pollution. Meanwhile, metal, alloy, and ceramic fibers are used in specific areas such as electronics, construction, and aerospace. However, their strong carbon footprints, high manufacturing cost and density restrict their wider applications toward a sustainable society.

Alternatively, natural fibers offer a more sustainable choice for developing functional fiber products. Silk [25–29] and plant fibers [30–33] are two excellent examples of natural fibers with advantageous features, such as biodegradability, renewability, low carbon footprint, and light weight. Although silk fibers display high mechanical strength (0.5–1 GPa in tensile strength), the low production volume and high manufacturing cost limit their wide applications. In contrast, natural-plant fibers (e.g., wood- and grass-based fibers) have greater availability and low-cost manufacturing; however, they generally feature poor mechanical performance due to the vessels and parenchymal cells showing a hollow and porous structure [34–36].

**Figure 1.**
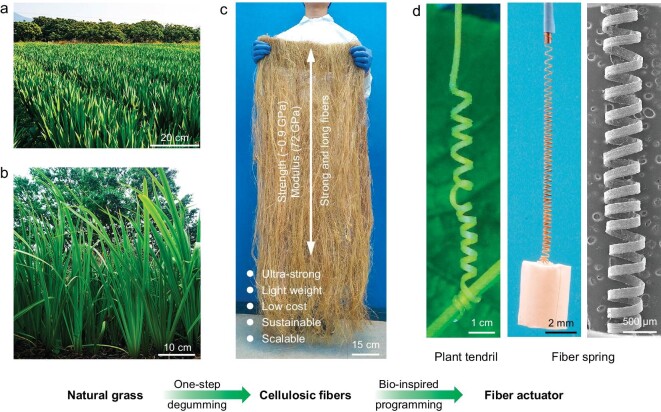
Strong, stiff, and smart cellulosic fibers. (a and b) Annual grass is abundantly available worldwide. (c) High-performance cellulosic fibers can be fabricated from natural grass using a simple yet effective degumming process, in which lignin and hemicellulose functioning as the glue are chemically removed, leading to the separation of the vessels and parenchymal cells and leaving behind cellulosic fibers. (d) The smart cellulosic fiber-based actuator. Inspired by the stretchable tendrils of natural plants, we can program the cellulosic fibers into a humidity-responsive actuator with a spring-like structure that controllably lifts and releases the attached objects.

Herein, we report a time-saving and cost-effective degumming strategy to manufacture mechanically strong, stiff, and humidity-responsive smart cellulosic fibers from fast-growing, abundant grass material at kilogram scale (Fig. 1a–c). Note that natural grass is generally composed of cellulosic fibers, vessels, and parenchymal cells, which are glued together by a matrix of lignin and hemicellulose into an integrated structure. In this one-step degumming process, lignin and hemicellulose are chemically removed, which enables the effective separation of the vessels and parenchymal cells, thus harvesting the cellulosic fibers from the natural grass. The resulting cellulosic fibers exhibit a dense morphology composed of the intact unidirectional cellulosic fibrils at both micro- and nano-scales, which is significantly different from the porous and heterogeneous structure of the natural grass starting material. Such densely packed aligned fibrils with strong interfibrillar interactions (hydrogen bonding and van der Waals forces) enable a high tensile strength of up to ∼0.9 GPa and a Young's modulus of up to 72 GPa of the resulting cellulosic fibers. Additionally, inspired by the stretchable tendrils of natural plants, we developed a humidity-responsive smart actuator by programming the cellulosic fibers into a precisely designed spring-like structure (Fig. 1d). The smart cellulosic fiber-based spring-like actuator can reversibly lift and release a load (100-times its own weight) by controlling the external environmental humidity. These strong and smart cellulosic fibers can be sustainably manufactured at extremely low cost and are fully biodegradable, providing both environmental and economic advantages compared with some commercial polymer- and metal-based fibers in the promising fields of soft robots, responsive textiles, and energy generation.

## RESULTS AND DISCUSSION

Natural grass features a heterogeneous structure composed of hollow vessels and parenchymal cells, as well as cellulosic fibers along the growth direction (Figs S1 and S2). In this structure, the porous vessels and parenchymal cells are mainly responsible for transporting water and nutrients, while the cellulosic fibers primarily provide structural support. While essential to living plants, as a material, the porous vessels and parenchymal cells essentially function as the structural defects [37,38], reducing the mechanical properties of the natural grass.

**Figure 2.**
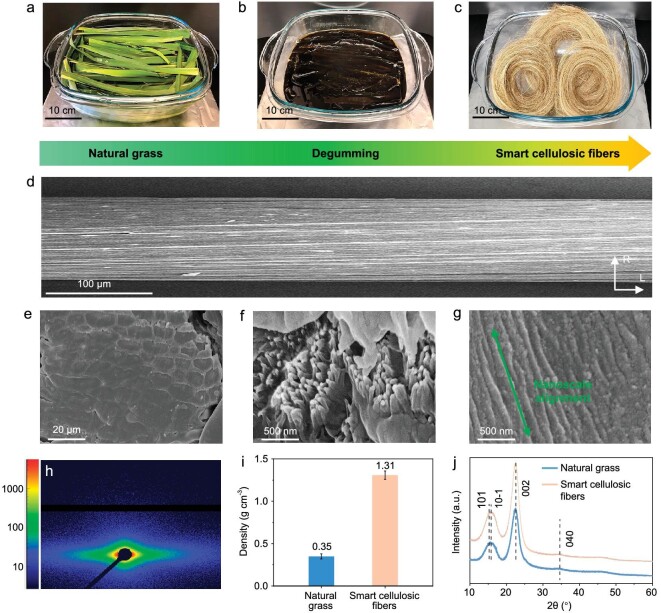
Synthesis and structural characterization of the grass-derived cellulosic fibers. (a–c) The cellulosic fibers can be facilely manufactured by a one-step degumming method of natural grass, where the adhesive of lignin and hemicellulose are chemically removed, followed by the separation of vessels and parenchymal cells, and leaving behind the cellulosic fibers. (d–g) The resulting cellulosic fibers feature a (d) smooth surface and (e) dense structure, as well as (f and g) unidirectional nanofibrils aligned along the fiber axis orientation. Specifically, the cellulosic fibers are composed of aligned microfibrils (e), which are further made up of aligned nanofibrils (f and g). (h) The small angle X-ray scattering (SAXS) pattern of the cellulosic fibers further demonstrates their aligned structure. (i) Density of the natural grass and resulting cellulosic fibers. (j) The cellulosic fibers present a typical cellulose I crystalline structure.

To improve the strength of the natural plant structure, we developed a degumming process, which involved using a mixture of NaOH and Na_2_SO_3_ to partially remove the adhesive of lignin and hemicellulose in the grass structure (see Methods for more details). Following the removal of the adhesives (lignin and hemicellulose), vessels and parenchymal cells easily detach from the main structure of the grass and disperse in the degumming aqueous solution. On this basis, the residual abundant cellulosic fibers can be harvested from the natural grass via removing the separated vessels and parenchymal cells (Fig. 2a–c). The resulting cellulosic fibers exhibit a relatively high cellulose content of 85%, with little hemicellulose (7.2%), and lignin (2.3%) compared to the natural grass (Figs S3 and S4). Fourier-transform infrared (FT-IR) spectroscopy also confirms the partial removal of hemicellulose and lignin, as indicated by the disappearance of the characteristic peaks of C=O and aromatic groups [39] following the degumming process (Fig. S5).

These prepared cellulosic fibers can be as long as ∼1 m with a dense structure composed of regularly stacked and compactly bonded cellulosic microfibrils (Fig. 2d and e; Figs S6 and S7). At a finer scale, we found numerous aligned nanofibrils parallel to the fiber axis orientation constituting the microfibrils (Fig. 2f and g; Fig. S8). Small angle X-ray scattering (SAXS) analysis further indicates the highly ordered alignment of the cellulosic fibrils in the cellulosic fibers (Fig. 2h). Due to the removal of the hollow vessels and parenchymal cells, as well as the amorphous hemicellulose and lignin, the resulting cellulosic fibers feature a high density of 1.31 g cm^−3^ (Fig. 2i), which is 3.7-times higher than the natural grass and even close to the density of nanocellulose (1.5 g cm^−3^) [30]. Thermogravimetric analysis (TGA) results of the cellulosic fibers also demonstrate slightly higher thermal stability compared with the natural grass (Fig. S9). In addition, the degumming treatment does not change the crystalline structure of cellulose I of grass (Fig. 2j).

**Figure 3.**
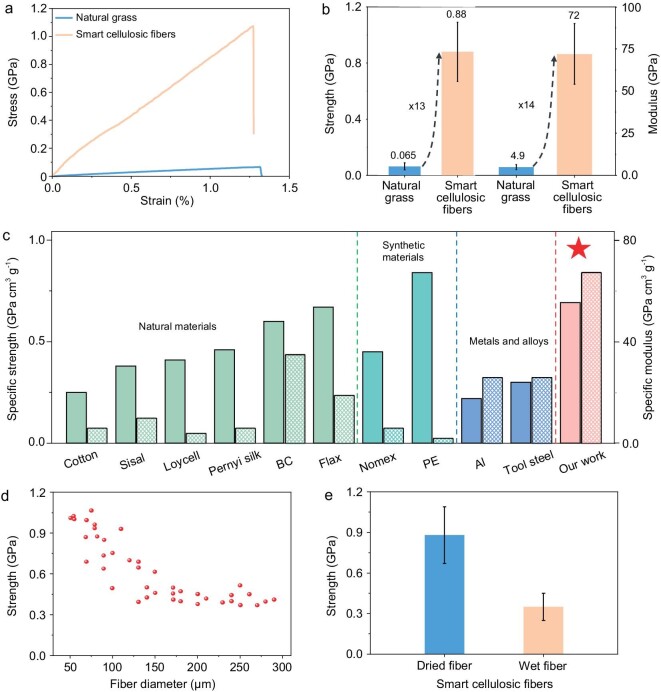
Mechanical properties of the cellulosic fibers. (a) Tensile stress-strain curves of the natural grass and cellulosic fibers. (b) The tensile strength and modulus of the natural grass and cellulosic fibers. The cellulosic fibers are stronger and stiffer compared to the natural grass. (c) The specific strength and specific modulus of the cellulosic fibers compared with other fibers made of natural materials, synthetic materials, and metallic alloys [2,43]. The solid and cross-hatched bar plots indicate the specific strength and specific modulus, respectively. (d) The effect of fiber diameter on the tensile strength of the cellulosic fibers. (e) The strength of the dried and wet cellulosic fibers.

We measured the mechanical properties of the dense and aligned cellulosic fibers. The stress-strain curves of the tensile tests are shown in Fig. 3a, in which the cellulosic fibers exhibit a higher maximum force with a similar elongation at break compared to the natural grass (Fig. S10). The cellulosic fibers feature an amazingly high average tensile strength of up to ∼0.9 GPa and Young's modulus of up to 72 GPa, which are 13- and 14-times higher than the original natural grass, respectively (Fig. 3b). We also investigated the fracture surfaces of the natural grass and cellulosic fibers after the tensile tests (Figs S11–S13). The natural grass composed of vessels, parenchymal cells, and fibers shows a non-uniform fracture surface (Fig. S11a). Brittle fracture with a smooth fracture surface occurs in the hollow vessels (Fig. S11b), indicating their role as structural defects in the material and the cause of the natural grass's weak strength [40]. In contrast, the cellulosic fibers featuring the dense and aligned fibrillar structure demonstrate a ‘pull-out’ fracture character under tensile stress (Figs S12 and S13). This type of aligned structure has been demonstrated as a crucial factor for promoting the mechanical performance of macrofibers [30,41,42]. Specifically, the compact stacking and interlocking of the cellulosic fibrils at both the micro- and nano-scales (Fig. 2e–g; Figs S6 and S8) reinforce the interfacial bonding (e.g., van der Waals forces and hydrogen bonds). Additionally, the dense and unidirectional fibrils can effectively transfer and decentralize the stress, thus enabling the super-strong mechanical performance of the cellulosic fibers. Note that strong degumming treatment weakens the mechanical strength of smart cellulosic fibers due to the alkaline degradation of cellulose molecules (Fig. S14).

We compared the mechanical strength performance of the cellulosic fibers with other natural fiber materials [2,43], including cotton, sisal, flax, lyocell, bacterial cellulose, and silk, in addition to synthetic fiber materials [2], such as Nomex and polyethylene (Fig. 3c). Notably, the cellulosic fibers exhibit a remarkable advantage of simultaneous high specific strength and specific modulus, which are much higher than those of previously reported fiber-based materials (Fig. 3c). In addition, the cellulosic fibers feature ∼2.5-times higher specific strength and specific modulus compared to some of the most commonly used structural materials, such as metals and alloys (e.g., Al metal and tool steel alloy) [2] (Fig. 3c). We also investigated the effect of the fiber diameter on its strength and found that larger-diameter cellulosic fibers show weaker strength compared to those with smaller diameters (Fig. 3d). Such diameter-dependent mechanical properties can be explained by the existence of a larger number of defects in the larger-diameter fibers. In addition, the cellulosic fibers present a wet strength of 0.35 GPa (Fig. 3e), which is superior to many cellulose-based fibers reported previously [44–46].

**Figure 4.**
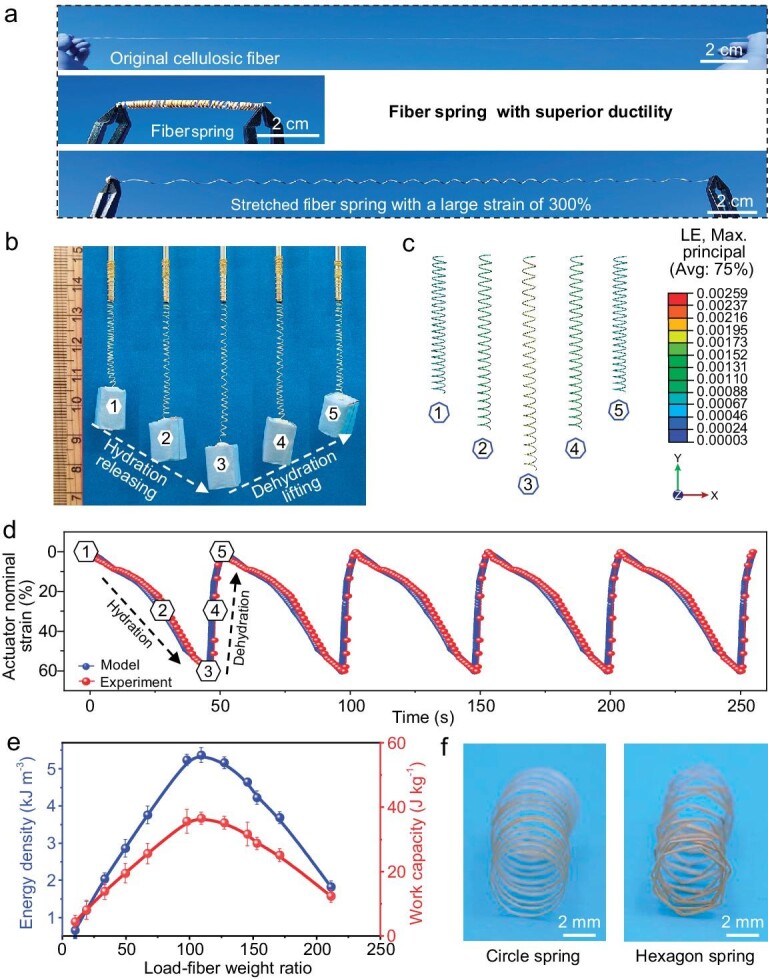
A humidity-responsive smart cellulosic fiber-based spring actuator. (a) The cellulosic fibers can be easily fabricated into a spring structure with superior ductility. (b) The cellulosic fiber can function as a humidity-responsive actuator to effectively release and lift an object. The hydration of cellulosic fiber takes place at room temperature (25°C) with 75% humidity, and its dehydration is realized at 50°C. (c) Modeling results show the variation of strain of cellulosic fiber spring functioning as the actuator, where the maximum principal strain of the cellulosic fiber is only 0.259% when it is elongated to a maximum nominal strain of 60%, much less than its failure limit. (d) The cellulosic fiber actuator can release and lift an object 100-times its own weight for multiple cycles, by designing the hydration and dehydration procedures. (e) The working output of the cellulosic fiber actuator, including the energy density and work capacity. (f) Different-shaped cellulosic fiber springs, including circular and hexagonal-shapes.

Inspired by the stretchable tendrils of natural plants, we developed a humidity-responsive smart actuator by fabricating the cellulosic fibers into a spring structure (Fig. S15). To fabricate the spring, we simply wound the wet cellulosic fibers on a metal rod, followed by drying to remove water. After releasing it from the metal rod, the cellulosic fibers retain the spring morphology, which can withstand a large elongation (Fig. 4a). Considering cellulose molecules feature abundant hydroxyl groups that are extremely sensitive to water molecules [47–49], we hypothesized the length of the cellulosic fiber spring would change as a function of the humidity. By designing the hydration and dehydration procedures, we can accurately control the displacement of the cellulosic fiber spring by tuning its stiffness. Previous works have experimentally demonstrated that the mechanical properties of cellulose-based fibers are sensitive to moisture [50–52]. An increase in moisture content decreases the stiffness/modulus of the cellulosic fibers, while a reduction in moisture content causes the reverse effect (Fig. S16). When adding a certain load within its linear elasticity limit, the cellulosic fiber spring first elongates to a certain position to reach the force equilibrium according to Hooke's Law. Then during the hydration process, the stiffness of the cellulosic fiber decreases as a function of the humidity, causing the spring to elongate further to accommodate the existing load in order to reach the force equilibrium again. During the subsequent dehydration process, the stiffness of the cellulosic fiber spring recovers to its original value, thus decreasing its elongation and moving back to the initial equilibrium position (Fig. 4b and Movie S1). In this manner, we were able to construct a humidity-responsive smart actuator that can effectively release and lift an object.

We further validated this actuation mechanism using finite element modeling (Fig. 4c and Movie S2). It is worth noting that when elongated to a maximum nominal strain of 60% of fiber spring actuator, the maximum principal strain in the cellulosic fibers is only 0.259% (Fig. 4c), which is far away from its failure limit of 1.23% in the dry state (Fig. 3a). This further demonstrates the linear elasticity of the cellulosic fibers during the whole actuation cycle, suggesting its potential in multi-cycle use. Figure 4d illustrates the time-dependence of the cellulosic fiber actuator strain during working cycles as we increased and decreased the ambient humidity (see Methods for details), thus hydrating and dehydrating the cellulosic fiber spring. When working at a load of 100-times cellulosic fiber weight, the response time for the hydration-driven releasing behavior was 45 s, and 5 s for the dehydration-induced lifting behavior. The difference in the response time for the hydration/dehydration-based releasing/lifting behavior of the cellulosic fiber actuator can be attributed to the fact that the actuator works at 50°C, which is beneficial for its dehydration process.

We also investigated the reversible releasing/lifting behavior of the cellulosic fiber actuator when loaded with different masses of weight. As expected, the maximum working strain negatively relates to the working load (Fig. S17). The maximum nominal strain of the cellulosic fiber actuator can reach up to 75% with a load of 10-times the cellulosic fiber weight loads at 40% working humidity. At a load of 100-times the fiber weight, the actuator outputs a 5.2 kJ·m^−3^ energy density and 35.7 J·kg^−1^ work capacity (Fig. 4e). Additionally, the cellulosic fiber actuator can be fabricated into different shapes, including circular and hexagonal structures (Fig. 4f). The diameter of the cellulosic fiber spring can also be controlled to construct actuators with a large range of deformability, where strain can be as high as 4000% (Figs S18 and S19). We envision that these strong and humidity-responsive smart cellulosic fibers could find a range of potential applications in textiles, smart robots, bio-composites, and energy generation fields.

## CONCLUSIONS

In summary, we demonstrated the simple, cost-effective and kilogram-scale fabrication of a strong, stiff, and humidity-responsive material from naturally abundant grass through a top-down degumming process. In such a process, lignin and hemicellulose functioning as adhesives are chemically removed, which leads to the effective removal of hollow vessels and parenchymal cells and harvesting of cellulosic fibers from the grass starting material. The cellulosic fibers featuring a dense, oriented, and uniaxial structure, are composed of stacked cellulosic fibrils at both micro- and nano-scales. The unique structure endows the cellulosic fibers with excellent mechanical properties, including a tensile strength of up to ∼0.9 GPa and a modulus of up to 72 GPa, which are among the highest values ever reported for various fiber materials, including natural, metal, ceramic, and synthetic polymer fibers. Inspired by the stretchable tendrils of natural plants, we further demonstrated a humidity-responsive smart actuator using the cellulosic fibers. The cellulosic fiber actuator shows a rapid response time of 50 s and a high lifting force of 100-times its own weight, translating to an energy density of 5.2 kJ·m^−3^ and a work capacity of 35.7 J·kg^−1^. These strong and smart cellulosic fibers, fabricated by a simple yet cost-effective degumming method using natural grass as a starting material, are a promising candidate for next-generation sustainable functional fibers for smart textiles, soft robots, bio-composites, and energy generation.

## METHODS

### Materials and chemicals

The natural gladiolus grass (Sesuvium portulacastrum) was harvested in Maryland, USA. Sodium hydroxide (>97%, Sigma-Aldrich), sodium sulfite (>98%, Sigma-Aldrich), and deionized (DI) water were used for processing the grass.

### Sample preparation

The natural grass was first soaked in water overnight. The wet grass was subsequently degummed by boiling in 2.5 M NaOH and 0.4 M Na_2_SO_3_ for 30 mins. Upon the completion of chemical treatment, the grass was fully washed with deionized water to remove the residual chemicals as well as the dissolved hemicellulose and lignin. Note that, due to removal of the glue of lignin and hemicellulose, the vessels and parenchymal cells can be also facilely removed during this process. The remaining fibers were separated and harvested, and air-dried for 24 h, to produce the final cellulosic fiber product.

### Characterization

The FT-IR spectrum was obtained by employing a Thermo Nicolet NEXUS 670 spectrophotometer at a wavelength of 500–4000 cm^−1^. The morphologies of the samples were observed using a field emission scanning electron microscope (SEM, Hitachi SU-70). Before the SEM observation, the samples were coated with Au for 300 s. The mechanical strength of samples was investigated by using an Instron 3365 universal tester according to the ASTM D3379-75 standard test method with an effective gauge length of 1 cm. Before testing, both ends of the samples were covered by soft sheets and then fixed to the instrument. The SAXS pattern was collected using a Rigaku MicroMax 007HF (operating voltage at 40 kV, current at 30 mA, Cu Kα, λ = 0.1541 nm). The compositional contents of the samples were measured using a two-step sulfuric acid hydrolysis process, as described previously [38]. XRD testing of samples was carried out on a Rigaku Ultima III equipped with a curved detector manufactured by Rigaku Americas Corp. The thermostability of the samples was measured using a thermogravimetric analyzer (SDT 650) at a heating rate of 5°C min^−1^ in air.

### Spring actuator

The cellulosic fibers were wetted with deionized water and subsequently wound on a metal rod, followed by drying to remove the water. After releasing the material from the metal rod, the cellulosic fibers retained the spring morphology, thus forming the spring actuator. To determine its humidity-responsive performance, the top end of the cellulosic fiber actuator was fixed to a copper rod using 3M tape, and its bottom end was connected to an object. A humidifier was employed to accurately control environmental humidity. The lifting/releasing processes of the actuator were recorded by a high-speed camera (Vision Research Phantom Miro M110) with a video recording speed of 3000 frames per second to quantify the performance of the humidity-responsive actuator. The actuator nominal strain, energy density, and work capacity were calculated following Equations (1), (2), and (3), respectively.


(1) \begin{eqnarray*}
\mathit{Nominal}{\mathrm{\ }}\mathit{strain}\ \left( \% \right) = \frac{{{{L}_t} - {{L}_0}}}{{{{L}_0}}}\times 100,
\end{eqnarray*}

(2) \begin{eqnarray*}
\mathit{Energy}\ \mathit{densty}\ ( {kJ\ {{m}^{ - 3}}} ) = \frac{{{{N}_o}*{{L}_o}}}{{{{S}_a}*{{L}_a}}},
\end{eqnarray*}

(3) \begin{eqnarray*}
\mathit{Work}\ \mathit{capacity}\ ( {J\ k{{g}^{ - 1}}} ) = \frac{{{{N}_o}*{{L}_o}}}{{{{m}_a}}},
\end{eqnarray*}

where *L_t_* and *L*_0_ are the actuator length at the working time of *t* s and 0 s, respectively in Eq. (1), *N_o_* and *L_o_* are the weight and distance moved by the object, and *S_a_* and *L_a_* are the cross-sectional area and length of the actuator, respectively in Eq. (2). *N_o_* and *L_o_* are the weight and distance moved by the object, respectively, and *m_a_* is the weight of the actuator in Eq. (3).

## Supplementary Material

nwae270_Supplemental_Files
