# Supplementary material for: Kilogram-scale production of strong and smart cellulosic fibers featuring unidirectional fibril alignment
Source: Natl Sci Rev. 2024 Aug 5;11(10):nwae270. doi: 10.1093/nsr/nwae270 (PMC11409887; doi:10.1093/nsr/nwae270)
Supplement: nwae270_Supplemental_Files [file nwae270_supplemental_files.zip › Supplementary data.pdf]

Supporting information

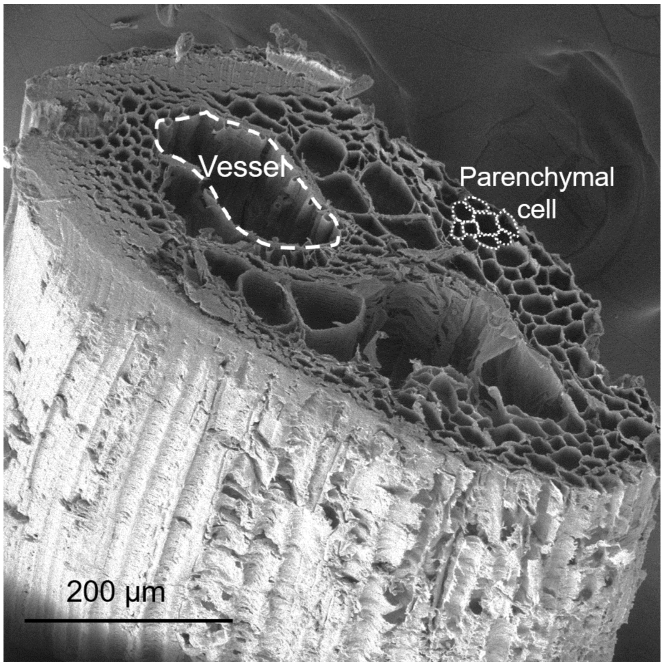

Figure S1. Cross-sectional SEM of the natural grass, which displays a heterogeneous structure that is composed of hollow vessels and parenchymal cells.

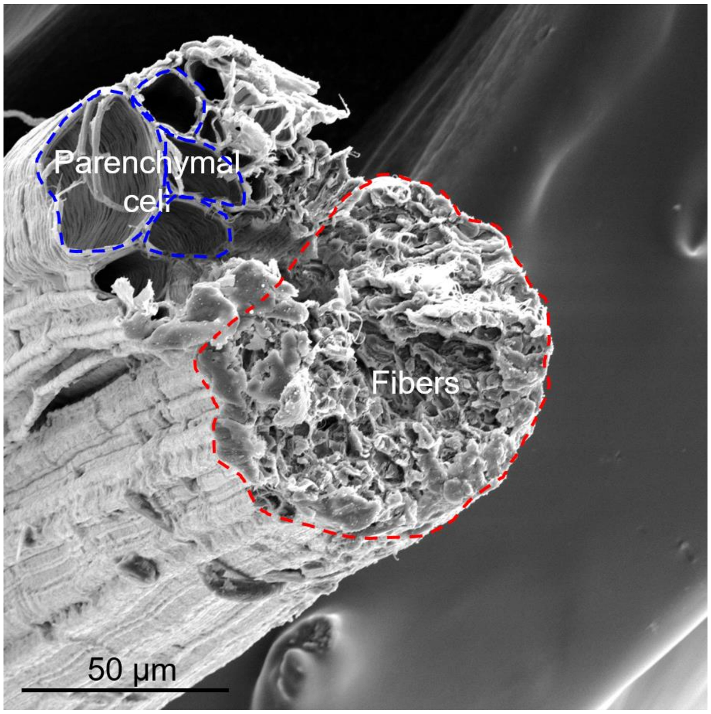

Figure S2. Cross-sectional SEM image of porous parenchymal cells and dense cellulosic fibers that compose the natural grass.

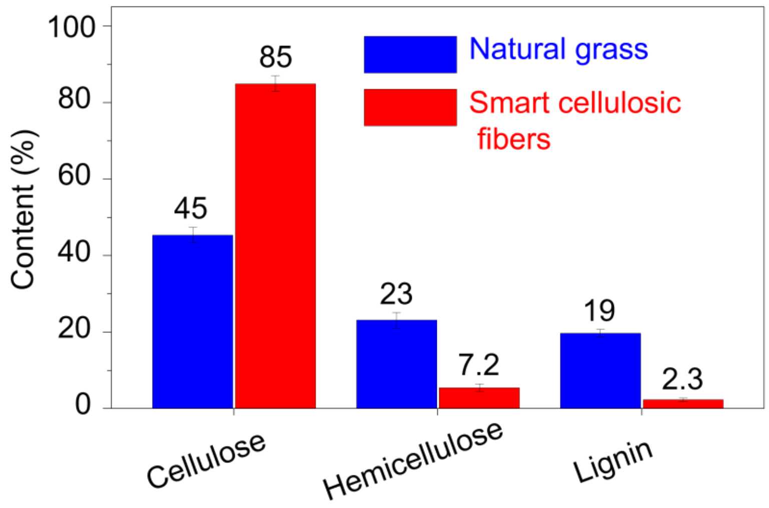

Figure S3. The chemical components of the cellulosic fibers and natural grass. The cellulosic fibers show increased cellulose content and lower hemicellulose and lignin contents compared to the natural grass starting material.

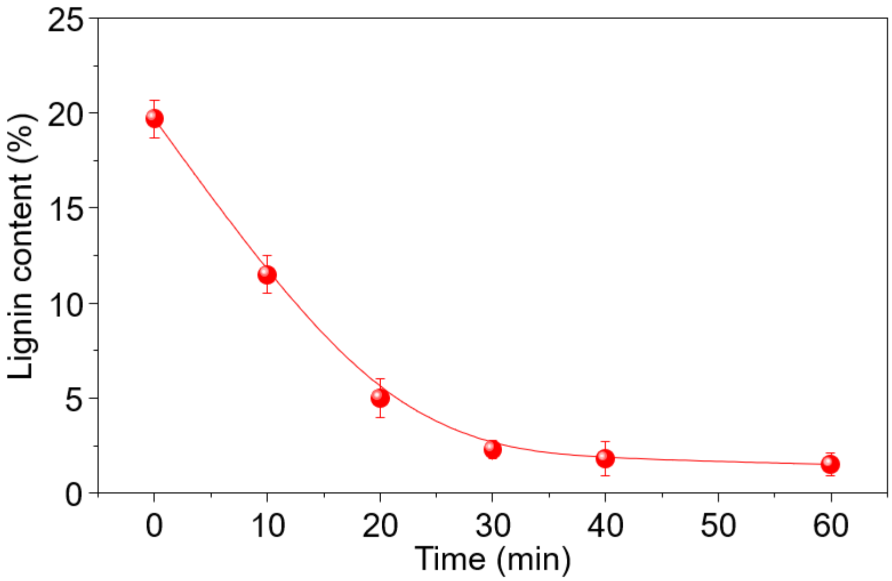

Figure S4. The lignin content as a function of the degumming time. The grass lignin can be removed rapidly, with its content decreasing to 2.3% after just 30 min treatment time.

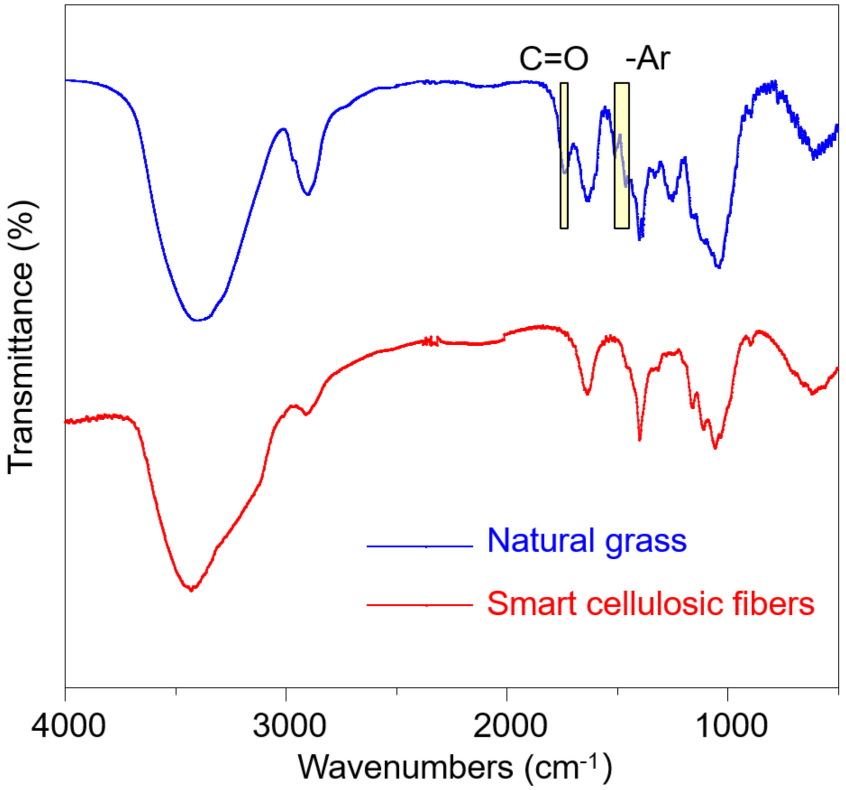

Figure S5. FT-IR spectrum of the natural grass and resulting cellulosic fibers, in which the functional groups associated with lignin and hemicellulose are almost completely disappeared after the degumming treatment.

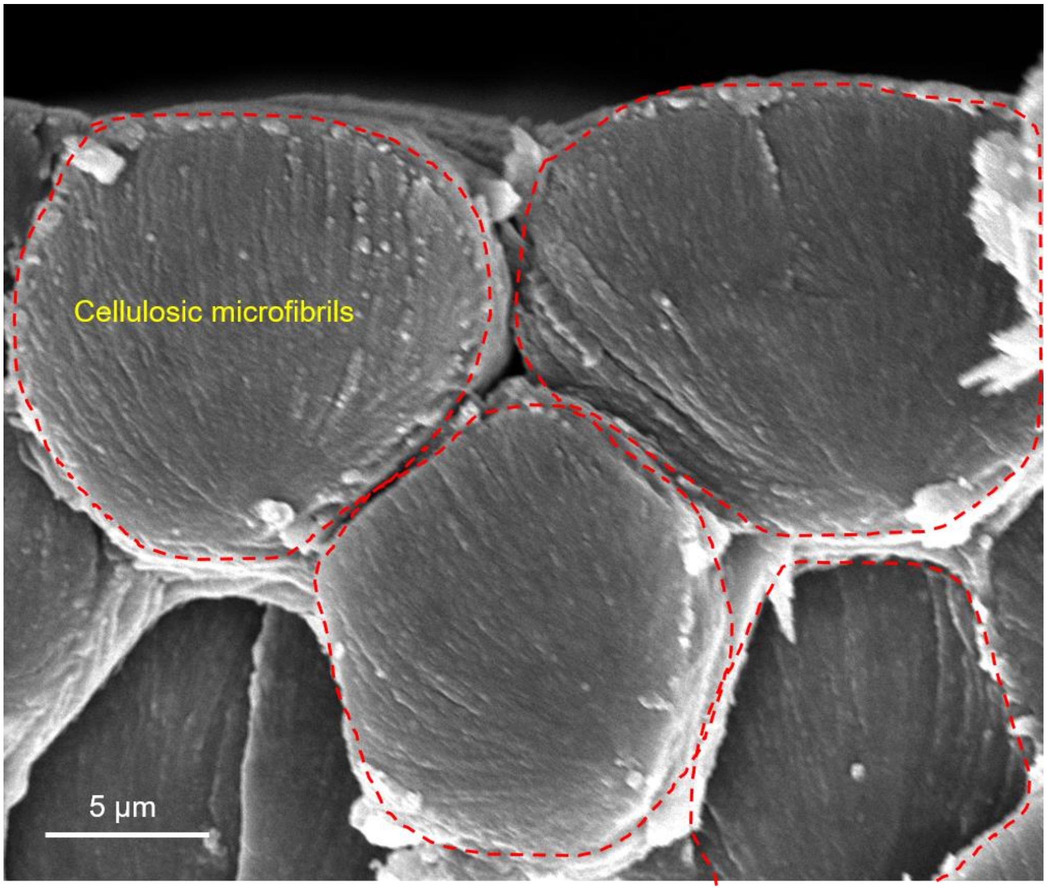

Figure S6. Cross-sectional SEM image of the cellulosic fibers after processing the natural grass starting material. The fibers feature a dense structure, in which the cellulosic microfibrils are closely stacked together.

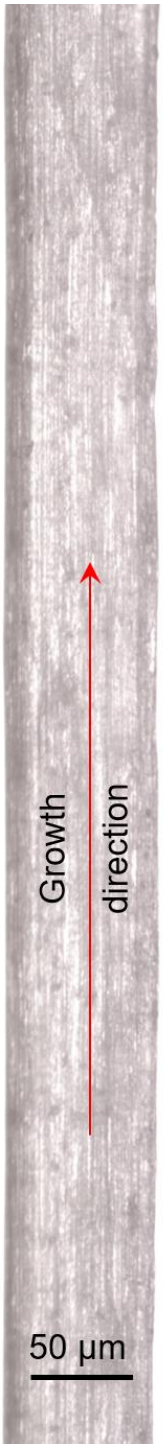

Figure S7. Optical microscopy image of the cellulosic fibers produced by the degumming treatment of the natural grass.

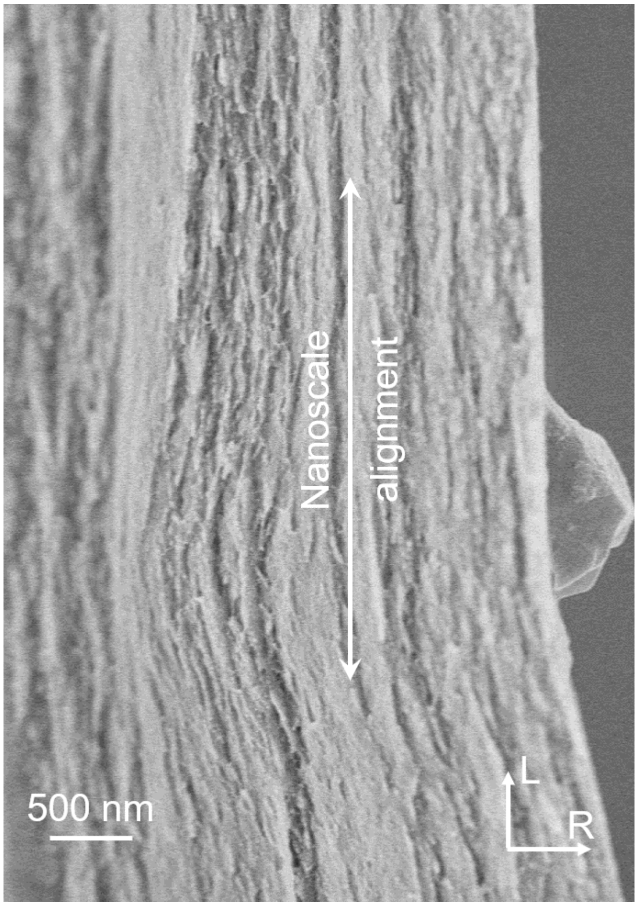

Figure S8. The nanofibrils of the cellulosic fibers. The cellulosic nanofibrils are assembled in a nanoscale lamellar structure that is highly oriented along the fiber axis direction.

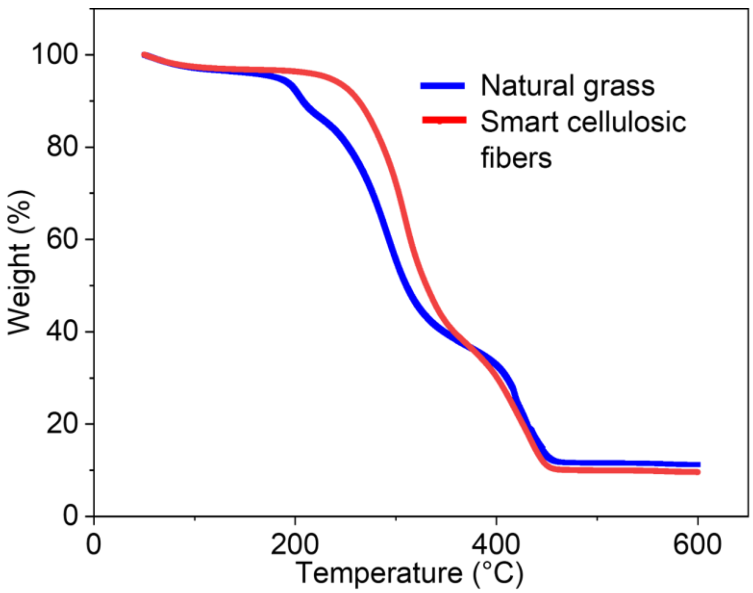

Figure S9. Thermal stability of the natural grass and cellulosic fibers. The cellulosic fibers exhibit a higher thermal stability compared to the natural grass starting material.

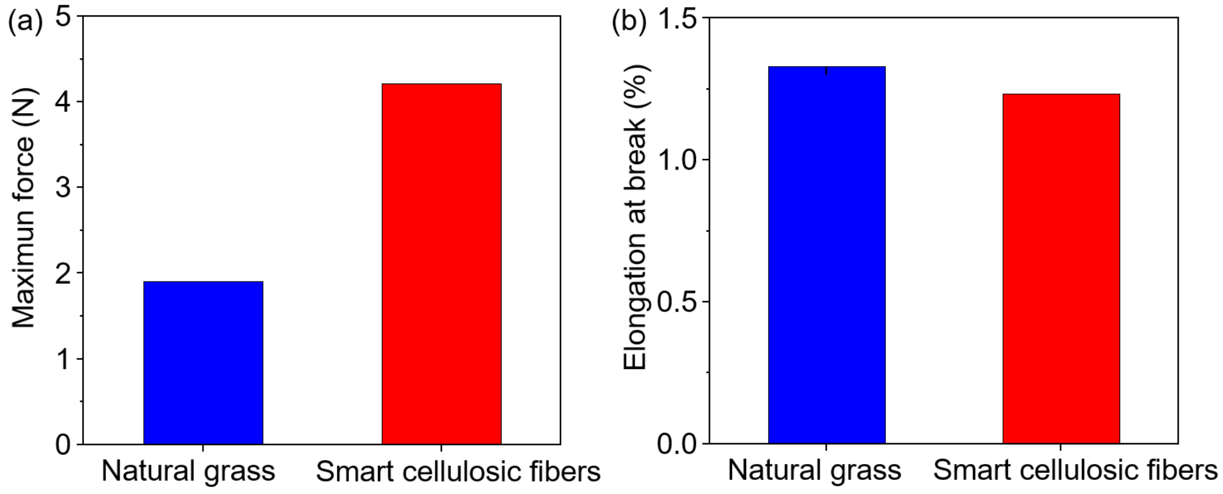

Figure S10. The (a) maximum force and (b) elongation at break of the natural grass and cellulosic fibers.

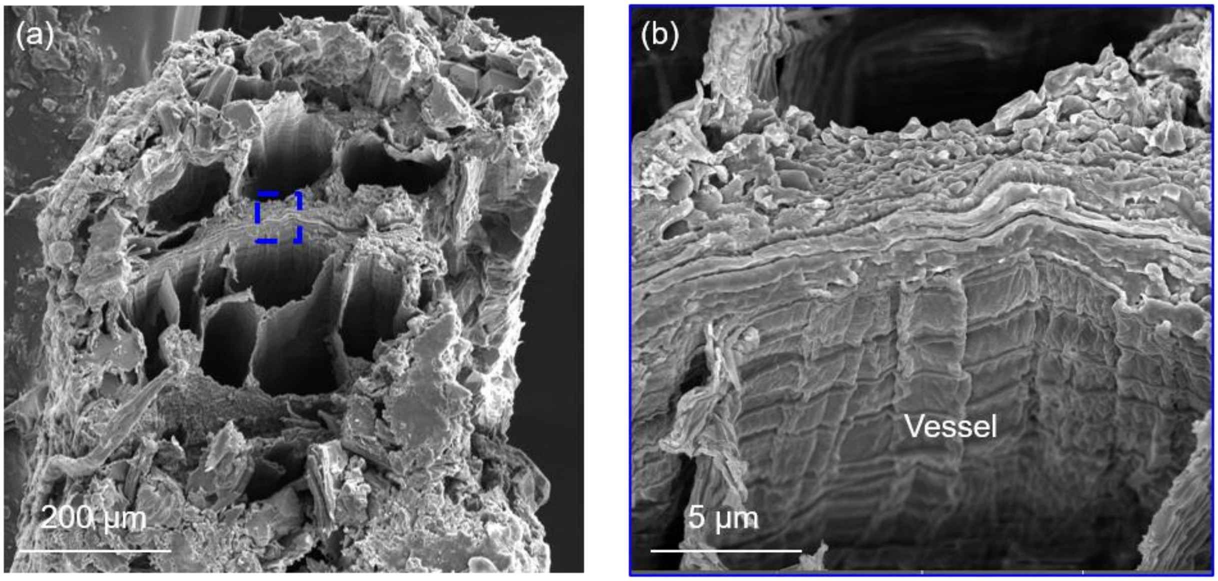

Figure S11. The fracture surface of the natural grass after tensile testing. The natural grass is composed of (a) hollow cells, in which (b) the vessels feature a smooth fracture surface.

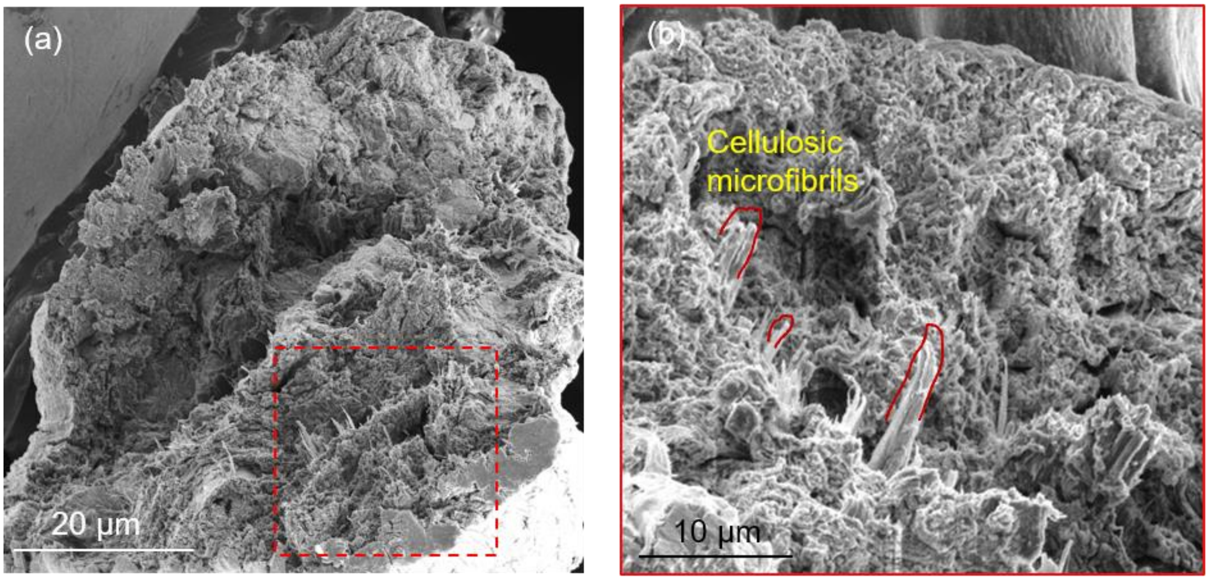

Figure S12. The fracture surface of the cellulosic fibers. (a) The dense cellulosic fibers display a large number of (b) cellulosic microfibrils on the fracture surface.

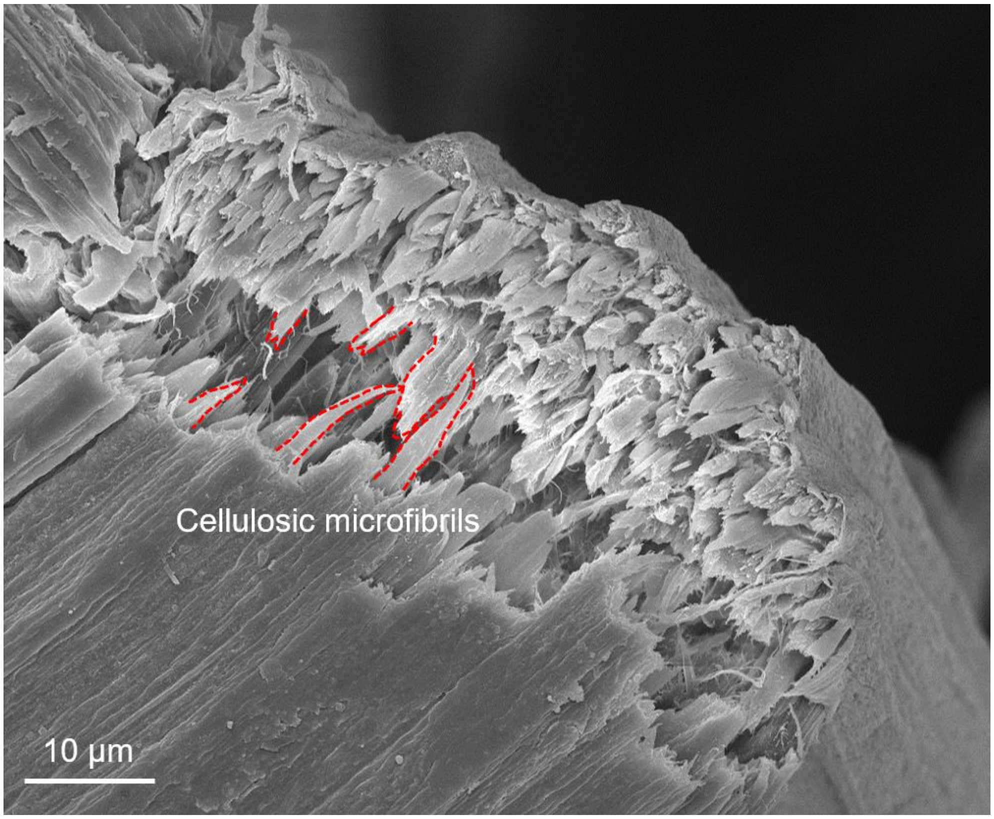

Figure S13. Fibrils at the fracture surface of the cellulosic fibers. The abundant cellulosic microfibrils compactly stack and bond with each other, thus endowing the cellulosic fibers with excellent mechanical strength.

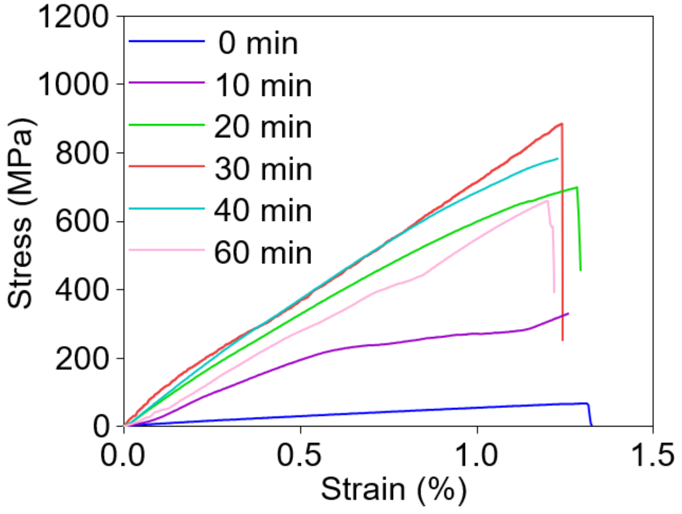

Figure S14. The mechanical strength of various cellulosic fiber samples as a function of degumming time.

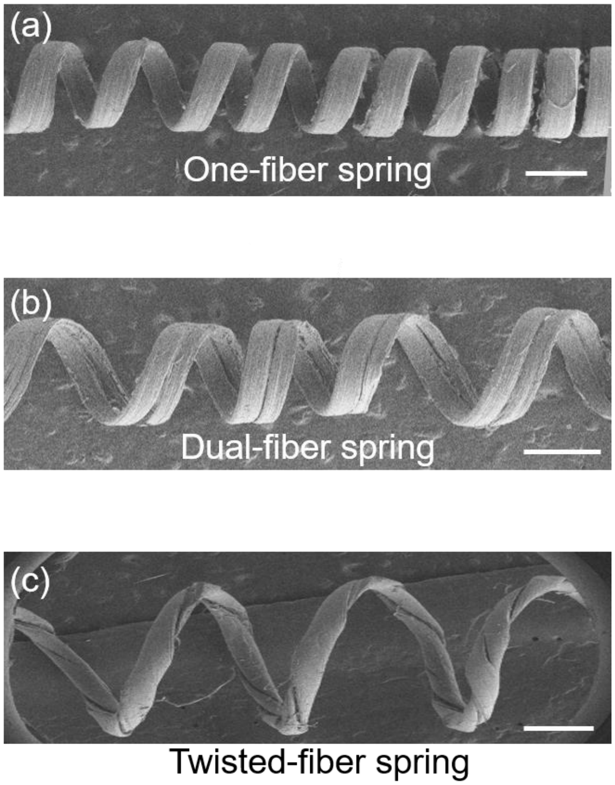

Figure S15. The cellulosic fibers were fabricated into different spring structures, including a common spring structure composed of a single fiber (a), a dual-fiber spring structure (b), and a twisted-fiber spring structure (c). Scale bar is 500 μm.

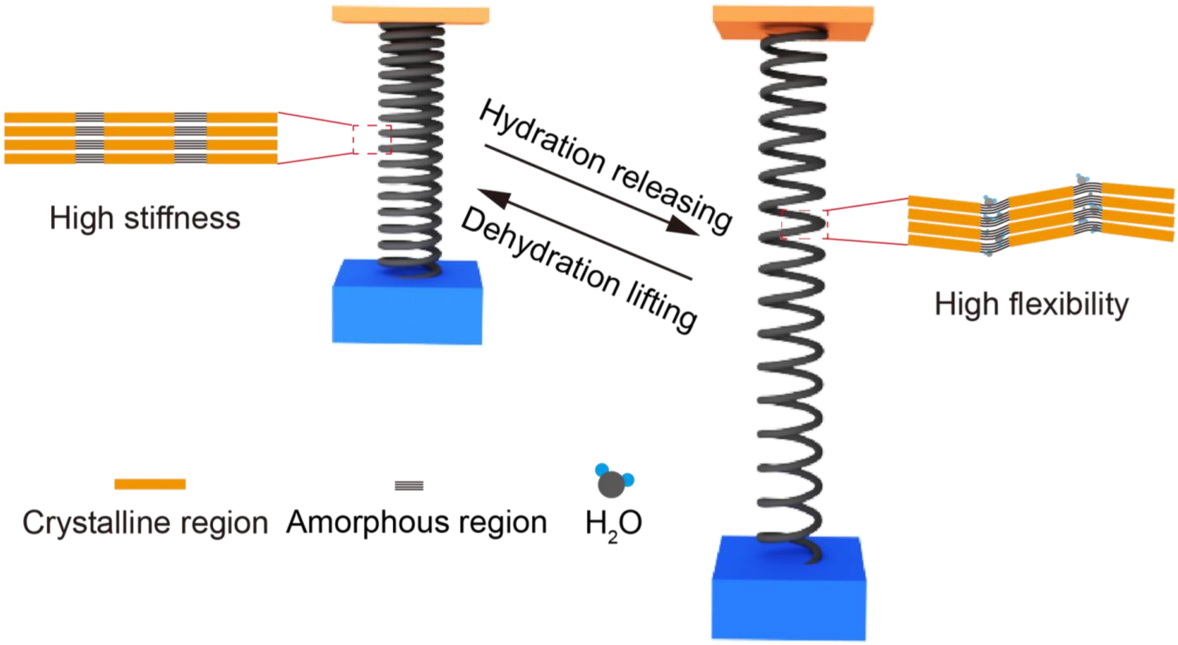

Figure S16. The schematic hydration-dehydration process of cellulosic fiber, where hydration treatment make cellulosic fiber flexible and dehydration treatment confers it with high stiffness.

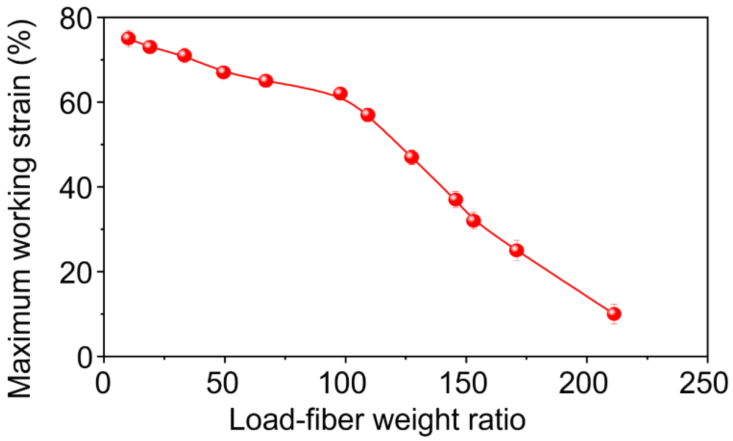

Figure S17. The actuator nominal strain when working with different loads at 40% working humidity. The maximum nominal strain can be 75% with a load of 10-times cellulosic fiber weight.

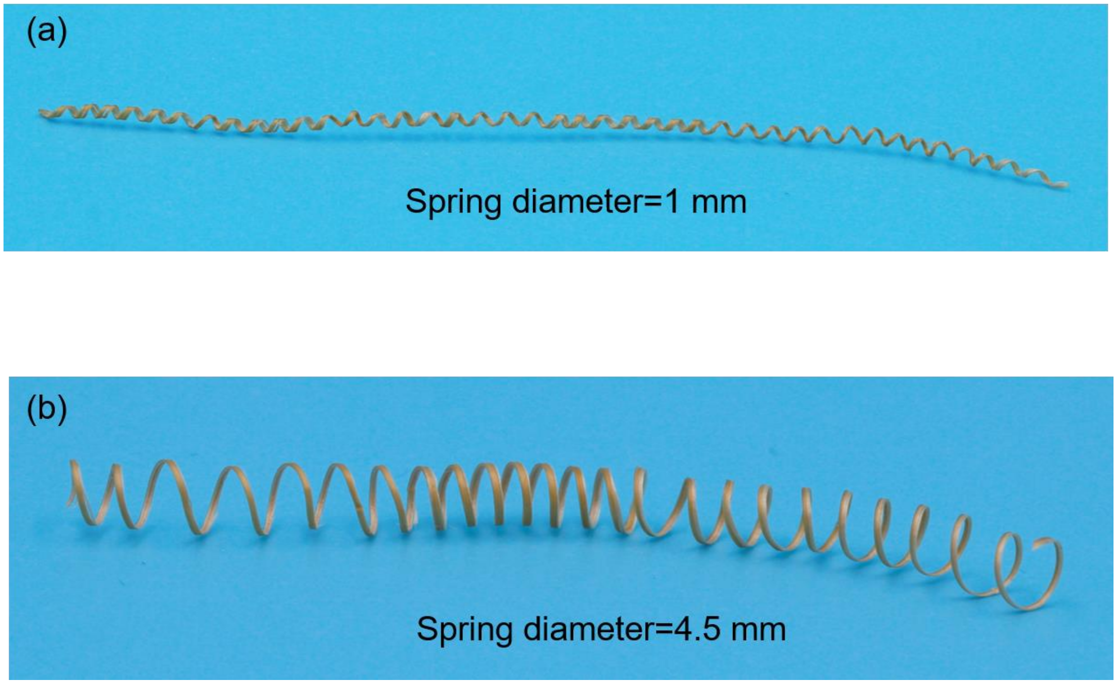

Figure S18. We can design cellulosic fiber springs with various diameters, including a diameter of (a) 1 mm and (b) 4.5 mm.

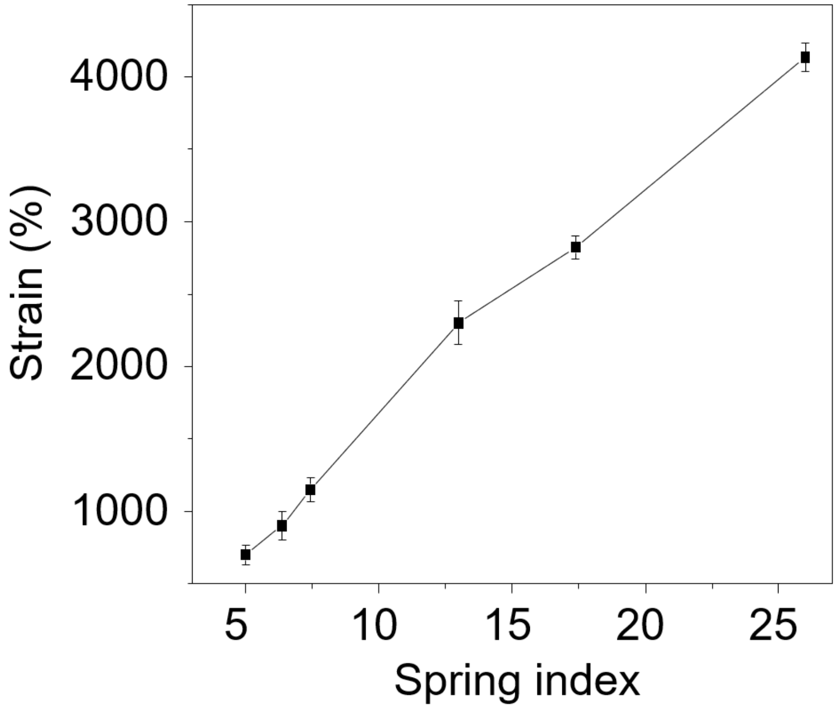

Figure S19. The actuator strain as a function of the spring index (the diameter ratio of actuator/fiber). We developed the actuators with variable spring index by controlling the diameters of the actuator and cellulosic fiber, in which the strain can be as high as 4000%.

Finite Element Modelling

The humidity-sensitive response of the cellulosic fiber spring actuator was computationally studied in the commercial finite element package ABAQUS/Standard 2021. The spring-patterned structure with a diameter of 2.3 mm was generated via repeating 21 times of a single helical unit along its axial direction. The geometry of the helical unit is determined by SEM image, with a unit length of 860 μm, fiber diameter of 110 μm, and thickness of around ¼ its diameter. The as-generated structures were meshed using 8-node thermally coupled brick, trilinear displacement and temperature elements (C3D8T) with an approximate global mesh size of 0.05 to ensure mesh quality. The Nlgeom setting is turned on considering the large deformation of up to 60% strain of the spring underwent.

Inherited from its anisotropic nature, the cellulosic fiber is regarded as a transversely isotropic material. In other word, all its mechanical properties in radial directions are equivalent to those in transverse direction, while different from those along the cellulosic fiber alignment. Detailed mechanical parameters of the cellulosic fiber spring actuator at different relative humidity used in simulation are shown in Table S1. A thermomechanical analysis was performed to simulate the response of the cellulosic fiber spring actuator to humidity change. The material properties of the cellulosic fiber are reasonably assumed dependent on relative humidity based on the experiment results in present and previous works.[1] By tuning the temperature boundary conditions, we realized the relative humidity variation as a function of time. All simulations were conducted in a fully coupled thermo-mechanical analysis.

Table S1. Humidity-dependent parameters used in mechanics modelling

**Relative Humidity (%)**	**40**	**54**	**62**	**67**	**71**	**75**

**E_L (GPa)**	72	69.1	67.2	62.2	59.4	55.8
**E_R, E_T (GPa)**	8.5	6.85	5.97	4.65	3.54	3.08
**G_{LT}, G_{LR} (GPa)**	4.90	3.95	3.44	2.68	2.04	1.78
**G_{RT} (GPa)**	3.27	2.63	2.30	1.79	1.36	1.18
**Poisson's ratio**			0.3			
**Density (g/cm^3)**			1.31			

*****Parameters along the longitudinal (the cellulosic fiber alignment) direction were determined from experiments results in present work, while parameters in transverse direction were assumed monotonically decreasing as the moisture content of grass fibers increased within a reasonable range from literature.[2, 3]

* E is Young's modulus; G is shear modulus; L, R, and T represents the properties along the longitudinal, radial, and transverse direction of the grass fiber, respectively.

References

[1] Yu M. Ultimate Strength characteristics of switchgrass stem cross-sections at representative processing conditions. Master's Thesis, University of Tennessee, **2004**.

[2] Sharma B, Jones, C, Khanchi A. Tensile strength and shear strength of switchgrass before and after frost. Biol. Eng. Trans. **2011**; 4:43-54.

[3] Dunn, G and Dabney S. Modulus of elasticity and moment of inertia of grass hedge stems. T. ASAE. **1996**; 39:947-952.
